# Building composite indices in the age of big data – Application to honey bee exposure to infectious and parasitic agents

**DOI:** 10.1016/j.heliyon.2023.e15244

**Published:** 2023-04-10

**Authors:** M. Huyen Ton Nu Nguyet, S. Bougeard, A. Babin, E. Dubois, C. Druesne, M.P. Rivière, M. Laurent, M.P. Chauzat

**Affiliations:** a Paris-Est University, ANSES, Laboratory for Animal Health, Maisons-Alfort, France; b ANSES, Ploufragan-Plouzané-Niort Laboratory, Epidemiology and Welfare of Pork, France; c ANSES, Sophia Antipolis Laboratory, Unit of Honey Bee Pathology, France; d ANSES, Research Funding & Scientific Watch Department, Maisons-Alfort, France

**Keywords:** Summarizing big datasets, Stress exposure, Honey bee health, Factor analysis, Synthetic indices

## Abstract

Pollinator insects play a crucial role in maintaining biodiversity and agricultural production worldwide. Yet they are subject to various infectious and parasitic agents (IPAs). To better assess their exposure to IPAs, discriminative and quantitative molecular methods have been developed. These tools produce large datasets that need to be summarised so as to be interpreted. In this paper, we described the calculation of three types of composite indices (numerical, ordinal, nominal) to characterize the honey bee exposure to IPAs in 128 European sites. Our summarizing methods are based on component-based factorial analyses. The indices summarised the dataset of eight IPAs quantified at two sampling times, into synthetic values providing different yet complementary information. Because our dataset included two sampling times, we used Multiple Factor Analysis (MFA) to synthetize the information. More precisely, the numerical and ordinal indices were generated from the first component of MFA, whereas the nominal index used the first main components of MFA combined with a clustering analysis (Hierarchical Clustering on components). The numerical index was easy to calculate and to be used in further statistical analyses. However, it contained only about 20% of the original information. Containing the same amount of original information, the ordinal index was much easier to interpret. These two indices summarised information in a unidimensional manner. Instead, the nominal index summarised information in a multidimensional manner, which retained much more information (94%). In the practical example, the three indices showed an antagonistic relationship between *N. ceranae* and DWV-B. These indices represented a toolbox where scientists could pick one composite index according to the aim pursued. Indices could be used in further statistical analyses but could also be used by policy makers and public instances to characterize a given sanitary situation at a site level for instance.

## Highlights

• The dataset of honey bee exposure to IPAs was summed up using three type of composite indices.
• Numerical, ordinal and nominal indices relied on factorial statistical methods.
• Pros and cons of the three composite indices were discussed using applications.
• The three indices showed an antagonistic relationship between *N. ceranae* and DWV-B.

## Graphical abstract

**Figure:**
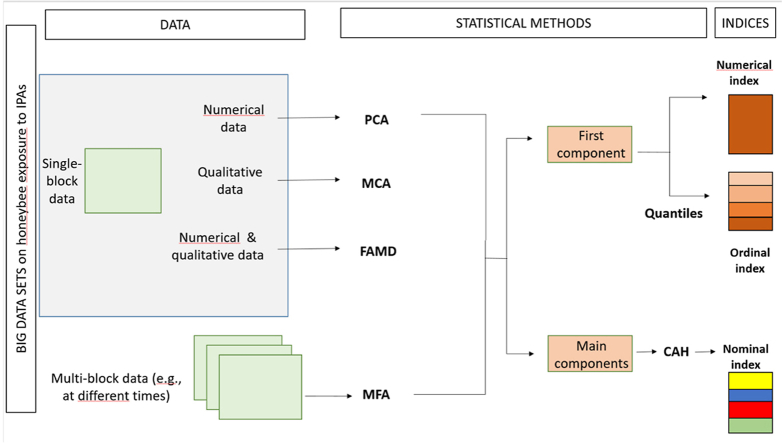

## 1 Introduction

Pollinating insects play a crucial role in maintaining biodiversity and agricultural production worldwide. Bees are the most important group of pollinators, visiting more than 90% of the leading 107 global crop types [1]. Honey bees (*Apis mellifera*) are the most used pollinators in agriculture. They are essential to 35% of the world's agricultural production, animal pollination services representing an annual economic contribution of up to 200 to 500 billion US dollars [2]. Under normal conditions, 10% is the acceptable level of colony winter losses for beekeepers [3]. However, the number of honey bee colonies in most European countries has been declining since 1965, and different causes for these losses have been proposed [4]. The intricate co-exposure to various stressors, such as infectious and parasitic agents (IPAs), chemicals released in the environment (i.e. insecticides, herbicides, fungicides), the depletion and poor quality of food sources, inappropriate beekeeping practices, and meteorological or climatic causes, as well as the interaction between them, weakens bee health and generates a worrying colony over-mortality. Consequently, studying the stress effects on bee health is becoming a crucial economic and ecological issue. The advent of new technologies made possible for biologists the collection of large datasets integrating information from multiple sources. Therefore, researchers need methods that summarise the information contained in these large datasets in order to perform statistical analyses and get meaningful insights from data.

Only a few methods are proposed in the literature to build indices for animal health. In most animal breeding, many models developed to characterize population health use methods that are either arbitrary [5] or not easily transferable to another animal species. Indeed, these models are tailored to or heavily dependent on the species characteristics [[6], [7], [8], [9]]. Especially in the bee world, in which most studies focused on honey bee populations, no previous work has reported the development of an index summarizing their exposure to multiple IPAs (the PRISMA bibliographic survey is detailed in Appendix A, supplementary material). Only two publications described a synthetic approach to explore the relationship between pesticide exposure and pathogen prevalence in honey bees [10,11]. The study of large datasets is possible using factor analyses, also known as component-based analyses [12,13]. These analyses are usually used as they provide synthetic graphical displays illustrating relationships between variables and between observations. Principal Component Analysis – PCA [12,14] or Multiple Correspondence Analysis – MCA [15,16] are some of the most popular methods of this family. However, factor analyses, and more precisely components sought from factor analyses, could also be used to seek indices.

In this paper, we focused our statistical analyses on IPAs. Our goal was not to include other factors (climate, landscape) in the indices. The proposed synthetic indices are not designed to describe multiple stressors affecting bees coming from a large dataset. We propose to apply a standard and transferable procedure based on factor analysis and adapted to the configuration of biological data, to seek an index, regardless of the nature and structure of the data. Depending on the needs of the biologists and/or the goal of communication, this index can be either numerical, ordinal (=ordered qualifier) or nominal (=unordered qualifier). These three types of indices were calculated on data of honey bee exposure to IPAs produced by the European project PoshBee on 128 sites (https://poshbee.eu/). We aimed at summarizing the several variables (i.e. eight IPAs quantified at two sampling times) into a single index that best described and discriminated the sites.

## 2 Materials and methods

### 2.1 Data

Data were produced in the frame of a Europe-wide project (PoshBee) which aimed at assessing, monitoring and mitigating stressors on bee health. PoshBee involved 14 European Union member countries over a five-year period (2018–2023). By “*developing a site network for assessing exposure of bees to chemical, nutritional, and pathogen stressors*” (Work Package 1), and by “*measuring chemical exposure, pathogens and aspects of nutrition in honey bees, bumble bees and solitary bees*” (Work Package 2), PoshBee provided several datasets with unique, robust and complete information [17]. Data were collected in 128 sites, with two focal crops, across eight European countries: Estonia (site numbers beginning with EST), Germany (GER), Ireland (IRL), Italy (ITA), Spain (ESP), Sweden (SWE), Switzerland (CHE), and United Kingdom (GBR). This means that eight sites per country for two crops were selected: 8 sites × 8 countries × 2 crops, leading to a total of 128 sites. At each site, three honey bee colonies were installed following the PoshBee protocols and over 50 measurements and samples related to the nutritional, toxicological, pathogenic and landscape components of the bees' environment were collected [18]. In the following list, we provide examples of collected information: data on the abundance and diversity of field margin floral resources (floral survey); survey of wild pollinators as an indicator of general ‘pollinator community health (pollinator survey); total and per hectare crop yields and inputs of agrochemicals (farmer survey) [18]. Adult honey bees were sampled in each colony and nest two times in 2019 for the detection and quantification of various IPAs at two sampling times: before bees were deployed on the sites (T0) and after bloom of the focal crop (T1). Bees from the three colonies and nests were pooled respectively. Ahead of the real-time qPCR analysis, samples were ground for nucleic acid extraction. Nucleic acids (RNA and DNA) were purified on spin columns (Machery-Nagel) with the high-throughput automated extraction of nucleic acids (TECAN pipetting robot). For both viruses and bacteria, standard qPCR protocols validated by the European reference laboratory for diagnosis (www.eurl-bee.anses.fr) were optimised with the reagents and the parameters for high-throughput qPCR [19]. For the three microsporidia species, a specific method was developed and validated using relevant molecular targets for an accurate quantification by real-time qPCR [20]. The loads of these IPAs were expressed in number of copies of the target IPA genome transformed into log_10_ per honey bee (log_10_ ge/bee).

The dataset contained 2805 observations, corresponding to the loads of 11 IPAs (initially 11 and subsequently reduced to 8 IPAs, see below) quantified at two sampling times (T0 and T1) on 128 sites: six viruses (Acute bee paralysis virus – ABPV, Black queen cell virus – BQCV, Chronic bee paralysis virus – CBPV, Deformed wing virus type A – DWV-A, Deformed wing virus type B – DWV-B, Sacbrood bee virus – SBV), the bacterial agents of the American foulbrood (*Paenibacillus larvae*) and the European foulbrood (*Melisococcus plutonius*) and three *Nosema* microsporidia (*Nosema apis*, *N. ceranae* and *N. bombi*). Qualitative variables which described the country and the focal crop were also included in the dataset. The theoretical number of analyses was 2816 (11 IPAs sampled in 128 sites at 2 times). After quality control, some samples were discarded (0.01%).

Amongst the 11 IPAs, *P. larvae*, *M. plutonius* and *N. bombi* were not detected in more than 90% of the samples at the two sampling times*.* Consequently, these three IPAs were not included in the development of the indices due to their very low presence (Table 1).

**Table 1:** Loads of eight IPAs (in log_10_ ge/bee) in honey bee samples collected in 128 PoshBee sites. The mean load was the mean of loads in samples collected at two times (T0 and T1).

IPA	Total number of samples analysed	Total number of positive samples (%)	Mean load	Mean standard deviation	Minimum load	Maximum load
**ABPV**	255	34 (13.3)	4.77	0.13	3.93	7.01
**BQCV**	255	254 (99.6)	7.61	0.18	4.68	10.88
**CBPV**	255	89 (34.9)	5.42	0.19	3.64	10.69
**DWV-A**	255	118 (46.3)	7.77	0.28	3.56	11.47
**DWV-B**	255	242 (94.9)	7.49	0.34	2.53	11.13
**SBV**	255	208 (81.6)	8.14	0.28	3.65	12.95
***N. apis***	255	45 (17.7)	7.07	0.09	5.71	8.12
***N. ceranae***	255	160 (62.6)	7.00	0.11	4.55	8.98

### 2.2 Statistical methods

It should be reminded that although the statistical methods presented here were not novel, this was the first time that these methods were used to characterize the exposure of bees to IPAs. This synthesis was a first step to interpret the exposure data. Subsequent steps may involve the use of structural equation modeling (SEM) for example.

The index was defined as a value assigned to a site, that summarises all the variables measured for that given site. Because components of factorial analyses are the best summary of several variables into a single one, these statistical methods were applied. Indeed, components are built as linear combinations of the variables obtained from a Singular Value Decomposition of the overall dataset [21]. Therefore, the first component explains the maximum amount of data variability.

The statistical procedure was organised in two steps. The first step consisted of reducing the dataset into a single (or into its main) component(s). The second step involved the calculation of the three indices from the first (or the main) component(s). Depending on the biologist's aim, three types of indices are proposed (numerical, ordinal or nominal). The first component was directly used to seek the numerical index, quantiles of the first component were used to seek the ordinal index, whereas several main components were used to seek the nominal index.

Step 1: Seek the first or main component(s) that best summarise the data. To summarise the large dataset into a single index, a factor analysis was performed and its first component was extracted.

For our data, the aim is to rank or classify into categories the 128 sites according to the bee exposure to the various IPAs. Since the variables are grouped into two sampling times (i.e. T0 and T1), a Multiple Factor Analysis (MFA) was applied [16].

Step 2: Process the index from the component(s). Three types of indices could be calculated and selected by the biologist depending on the aim pursued:

(i) a numerical index directly obtained from the first MFA component. It was the best summary of the whole dataset into a single dimension, the one that explained the greatest variability of the data;
(ii) an ordinal index from the quantiles of the first MFA component. This index was a transformation of the numerical index into ordinal levels using quantiles;
(iii) a nominal index produced from a clustering analysis (Hierarchical Clustering on Principal Components – HCPC [14]) applied to the first main MFA components. This method gave the best multidimensional summary of the whole dataset. The choice of the final number of clusters to be retained was discussed among statistical, biological and bee health experts.

The MFA method was applied to the eight IPAs measured at two different times. Country and focal crop of each site were considered as supplementary variables in the analysis. They were not used to process the components but only for additional interpretation.

The factor analyses were implemented in the R software version 4.1.2 (R Core Team, 2017) from the FactoMineR package [22].

## 3 Results: application to honey bee exposure to IPAs

### 3.1 Calculation and interpretation of the numerical index

**Fig. 1:**
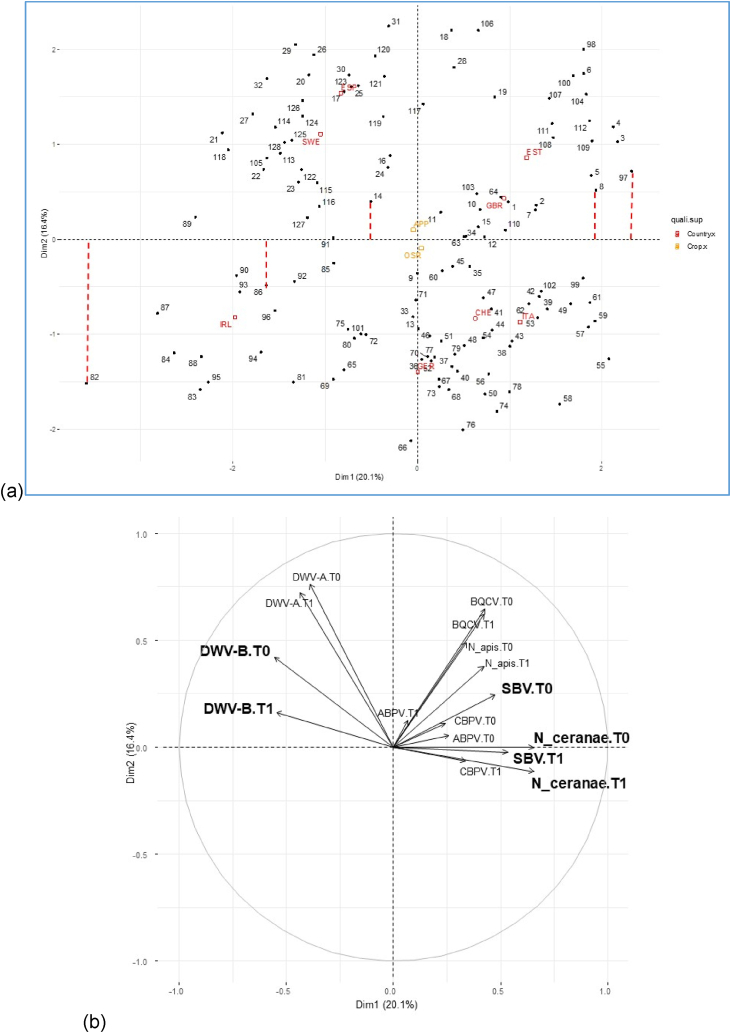
MFA results for the first two components of honey bee exposure to IPAs. (a) 128 PoshBee sampling sites representation (e.g., projections in red for the sites 82, 86, 14, 8 and 97). The numerical index corresponded to the projection of the sites on the first component. The two nominal supplementary variables (i.e., country and crop) were added. (b) Correlation circle of the IPA variables (i.e., 8 IPAS at the two times T0 and T1). The IPAs with the highest contribution (i.e., correlation >0.5) were highlighted in bold. (For interpretation of the references to colour in this figure legend, the reader is referred to the Web version of this article.)

The coordinates of the 128 sites on the first component of the MFA (horizontal axis) corresponded to the numerical index values (Fig. 1a and Appendix C). The index ranged from −3.58 for the 82nd site to 2.32 for the 97th site, with a mean value of 0 (*sd* = 1.31). It explained 20.1% of the total data variability (Appendix B). The IPAs DWV-B, SBV and *N. ceranae* were those which mostly contribute to build this component/index (Fig. 1b). In other words, the degree of exposure to IPAs that differentiates the 128 sampled sites was mostly characterised by these three IPAs. Moreover, the first component correlated congruently with the IPAs load on both sampling time, but not with the focal crop (Fig. 1). Therefore, no interpretation concerning the focal crop would be made, and unless mentioned otherwise, all interpretations applied to either modality of the group variable (T0 and T1).

To extract the numerical index values, the site coordinates were projected onto the first component, as illustrated in red for 5 out of 128 sites. Using the numerical coordinates, sites were ranked according to their exposure to the most contributing IPAs. Insights into the characteristics of each site were obtained based on a juxtaposition of the representation of sites (Fig. 1a) and variables (Fig. 1b): for example, sampled sites with negative coordinates (so-called “low index values”) were characterised by a higher load of DWV-B and lower load of SBV and *N. ceranae* compared to the sites that have an index value around 0; whereas sites with positive coordinates (so-called “high index values”) had a lower load of DWV-B and higher loads of SBV and *N. ceranae.*

This index can also be interpreted with the supplementary variable ‘country’ (Fig. 1a). The low-index-value sites were particularly common in Ireland, Spain and Sweden whereas the high-index-value sites were commonly found in Estonia, Italy, Switzerland and the United Kingdom. Most sites with a value around 0 were from Germany.

### 3.2 Calculation and interpretation of the ordinal index

In order to simplify the index interpretation, the latter numerical index could be transformed into an ordinal one by using quartiles for instance. The first quartile (Q1) corresponded to the limit below which 25% of the data is set. The second quartile (Q2) was the median (i.e., 50% of the data lies below this value). The third quartile (Q3) was the limit below which 75% of the data is set. By dividing the 128 sites into four equal quartiles (32 sites each), according to the relationships between each site coordinates and the cut-off value of each quartile, an ordinal index ranging from one to four was obtained. The first ‘site’ quartile clustered sites whose coordinates on the first component were strictly inferior to Q1 = −1.10. The second quartile clustered sites whose coordinates were comprised between Q1 = −1.10 and Q2 = 0.21. Sites with coordinates between Q2 = 0.21 and Q3 = 0.96 belonged to the third ‘site’ quartile and the last ‘site’ quartile grouped the remaining sites (Appendix C).

**Fig. 2:**
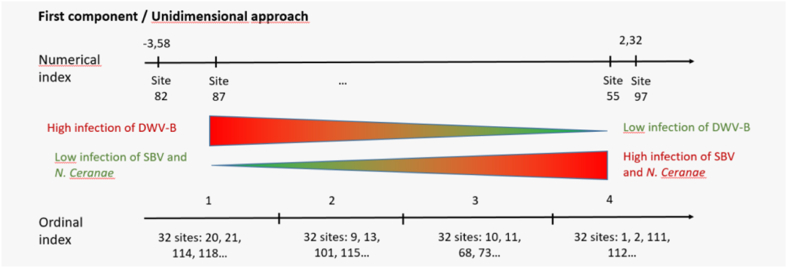
The numerical and ordinal synthetic indices calculated to summarise the exposure of honey bees to IPAs using the same dataset.

The meaning behind the score and each configuration of health status was the same as that of the numerical index. The higher the score, the lower the loads of DWV-B and the higher SBV and *N. ceranae* loads (Table 2 for the main features illustrated in Fig. 2).

**Table 2:** Ordinal index: median and inter-quartile range [Q1, Q3] of IPA loads (log_10_ ge/bee) at T0 and T1 in honey bees sampled in the 128 sites. Distribution of sites per country and crop in each quartile.

Levels	1 (n = 32)		2 (n = 32)		3 (n = 32)		4 (n = 32)	
Sampling times	T0	T1	T0	T1	T0	T1	T0	T1
**ABPV**	0 [0, 0]	0 [0, 0]	0 [0, 0]	0 [0, 0]	0 [0, 0.98]	0 [0, 0]	0 [0, 0]	0 [0, 0]
**BQCV**	6.77 [5.95, 7.70]	7.24 [6.69, 8.41]	6.66 [6.22, 7.41]	7.67 [7.20, 9.07]	7.01 [6.49, 7.98]	7.77 [7.35, 9.23]	7.94 [7.07, 9.12]	8.94 [7.71, 9.66]
**CBPV**	0 [0, 0]	0 [0, 0]	0 [0, 5.01]	0 [0, 4.38]	4.80 [0, 5.72]	4.46 [0, 5.18]	0 [0, 5.13]	0 [0, 5.10]
**DWV-A**	8.14 [0, 10.4]	8.18 [5.80, 9.59]	0 [0, 10.1]	1.78 [0, 7.80]	0 [0, 1.25]	0 [0, 5.99]	0 [0, 5.29]	0 [0, 4.89]
**DWV-B**	10.1 [9.56, 10.3]	9.29 [7.94, 9.91]	9.91 [8.49, 10.5]	9.10 [7.79, 9.90]	7.28 [5.85, 9.03]	8.01 [5.41, 9.42]	5.68 [4.25, 6.56]	4.25 [2.97, 5.68]
**SBV**	5.34 [0, 7.21]	0 [0, 9.29]	6.00 [5.21, 7.75]	8.84 [6.92, 11.1]	6.56 [5.88, 8.45]	8.96 [7.52, 10.4]	7.96 [7.04, 9.99]	10.3 [8.48, 11.0]
***N. apis***	0 [0, 0]	0 [0, 0]	0 [0, 0]	0 [0, 0]	0 [0, 0]	0 [0, 0]	5.84 [0, 7.28]	0 [0, 7.19]
***N. ceranae***	0 [0, 0]	0 [0, 0]	5.38 [0, 6.93]	6.29 [0, 6.75]	7.03 [6.69, 7.50]	6.88 [6.44, 7.15]	7.32 [6.85, 7.65]	7.13 [6.51, 7.82]
Country > 20% N (%)	IRL14 (44%) SWE 9 (28%) ESP 8 (25%)		GER 9 (28%) SWE 7 (21%)		CHE 9 (28%) ITA 7 (21%)		EST 11 (34%) GBR 8 (25%) ITA 8 (25%)	
Crop N (%)	Apple 18 (56%) Oil seed rape 14 (44%)		Apple 14 (44%) Oil seed rape 18 (56%)		Apple 19 (59%) Oil seed rape 13 (41%)		Apple 13 (41%) Oil seed rape 19 (59%)

### 3.3 Calculation and interpretation of the nominal index

The first step consisted in selecting the number of components of the MFA to be included in the clustering. This step led to (i) take into account the block structure of the data (T0 block and T1 block), and (ii) use the relevant part of the information (and avoid noise) while selecting components. According to the decrease of inertia (Appendix B), 12 components (out of 16, the total number of IPAs for the two times T0 and T1), for a cumulative 94% of the data inertia, were a relevant selection of the information contained in the data.

The second step consisted in selecting the best number of clusters. To do this, examination of criteria such as maximum loss of intra-class inertia and Ward's criterion suggested two-, three- or four-class clustering. According to the biologists' choice in relation with interpretation, a four-class nominal index was interpreted.

**Fig. 3:**
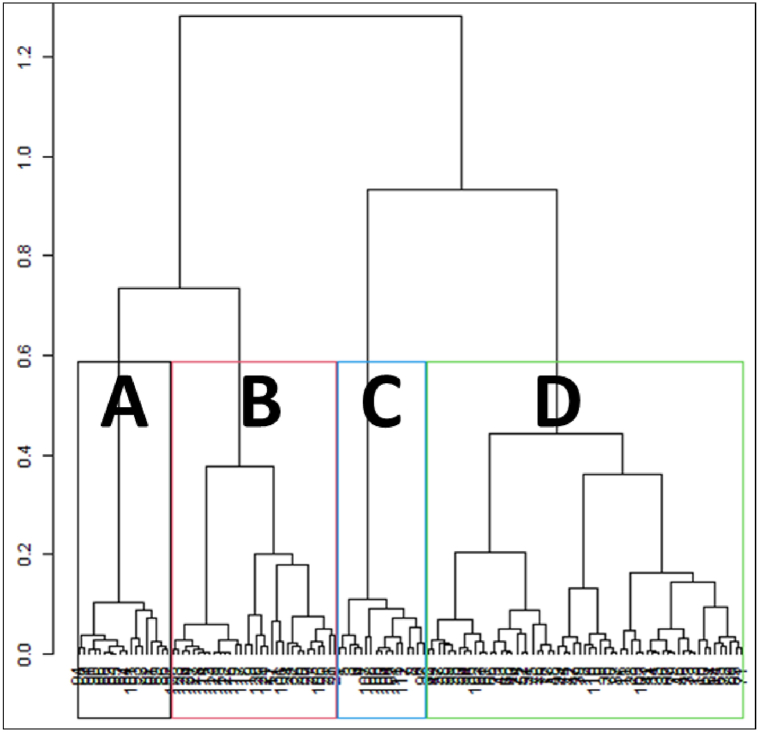
The 128 sites according to the first 12 MFA components. The four clusters renamed with a letter (see the text for description).

The nominal index was characterised mostly by the load of all the IPAs of interest at both sampling times. To emphasise the non-ordinal characteristics of the clusters, they were renamed with a letter instead of the default number (Fig. 3). Table 3 (and Appendix C) summarised the characteristics of the clusters as well as the distribution per country of the sampling sites in each cluster.

**Table 3:** Nominal index: mean of IPA loads (log_10_ ge/bee) at T0 and T1 in honey bees sampled in the 128 sites. Distribution of sites per country and crop in each cluster.

Levels	Overall mean		A (n = 19)		B (n = 34)		C (n = 57)		D (n = 18)	
Sampling times	T0	T1	T0	T1	T0	T1	T0	T1	T0	T1
ABPV	0.71	0.57	0	0	0.35	1.05 *	1.07 *	0.64	1.03	0.01
BQCV	7.29	8.13	5.87 *	6.78 *	7.89 *	8.68 *	6.91 *	7.91	8.84 *	9.20 *
CBPV	2.05	1.81	0 *	0.25 *	2.45	1.34	2.78 *	2.69 *	1.12	1.55
DWV-A	3.70	3.77	2.52	3.52	9.78 *	9.01 *	0.52 *	0.93 *	3.54	3.16
DWV-B	7.75	7.21	8.67	8.07	9.78 *	8.77 *	6.57 *	6.71	6.66	4.95 *
SBV	6.26	7.55	1.47 *	1.57 *	7.10	7.18	7.06 *	9.41 *	7.21	8.66
N. apis	1.48	1.05	1.39	0.70	0.93	0.19 *	0 *	0 *	7.32 *	6.35 *
N. ceranae	4.14	4.71	0 *	1.39 *	2.57 *	3.07 *	5.56 *	6.35 *	6.99 *	6.12
Country >20%			IRL16 (84%)	ESP 16 (47%)	CHE 16 (28%)	EST 10 (56%)				
N (%)				SWE 16 (47%)	ITA 16 (28%)	GBR 8 (44%)				
					GER 15 (26%)					
Crop			Apple 9 (47%)	Apple 18 (53%)	Apple 31 (54%)	Apple 6 (33%)				
N (%)			Oil seed rapes 10 (53%)	Oil seed rapes 16 (47%)	Oil seed rapes 26 (46%)	Oil seed rapes 12 (67%)				

* Significant difference between the value and the overall mean in the corresponding sampling time (T0 or T1) at threshold 0.05.

The clusters A, B, C, and D included respectively 19, 34, 57 and 18 sites. Both clusters A and B had significantly lower loads of *N. ceranae* compared to the average load of all sites. Cluster A was mostly characterised by significantly lower loads of BQCV, CBPV and SBV than the average load. Cluster B had significantly higher loads of ABPV, BQCV, DWV-A and DWV-B at T1, as well as a significantly lower loads of *N. apis* at T1. Cluster C represented the majority of the sampled sites. As opposed to clusters A and B, it had significantly higher loads of ABPV, CBPV, SBV and *N. ceranae* at T0 and of CBPV at T1 than the average. Cluster C was also characterised by significantly lower loads of DWV-A and DWV-B at T0 and *N. apis* at both sampling times. The last cluster (D), was characterised by significantly higher loads of BQCV and *N. apis* at both sampling times and *N. ceranae* at T0, as well as significantly lower loads of DWV-B at T1 than the average. In summary Cluster C and D were characterised by high loads of *N. ceranae* as opposed to clusters A and B.

**Fig. 4:**
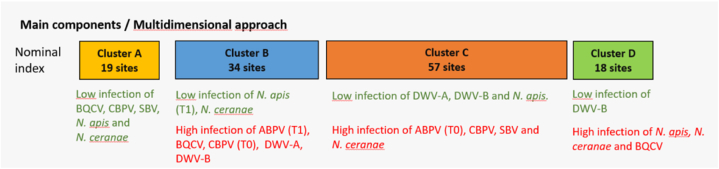
Nominal synthetic index calculated to summarise the exposure of honey bees to IPAs using the same dataset as the numerical and ordinal indices.

Interestingly, the country of origin was significantly related to the clustering as some countries were over-represented in some clusters (Table 3 for the main features illustrated in Fig. 4). Cluster A grouped mostly Irish sites, while cluster B was dominated by Spanish and Swedish sites. The majority of sampled sites were found in cluster C with sites from Germany, Italy and Switzerland. The majority of British and Estonian sites were identified in cluster D.

Overall, we found the same antagonistic relationship between *N. ceranae* and DWV-B across these four clusters as observed for the numerical index (Fig. 4). However, the information was completed with more nuances found for the six remaining IPAs as 94% of the information was contained in this index (instead of 20% for the numerical index). Among low-index-value sites, Ireland differed from Spain and Sweden in having an overall lower load of BQCV while the prevalence was rather high in those sites (cluster B). Among high-index-value sites, Estonia and United Kingdom differed from the other sites with a higher prevalence of *N. apis*. In addition, nuances related to the sampling times were better highlighted with the nominal index than with the numerical or the ordinal ones. A summary table (Appendix C) gave a full disclosure of the three types of indices for each PoshBee site.

## 4 Discussion and conclusion

In this paper, we described the calculation of three types of synthetic indices to characterize the honey bee exposure to IPAs. Depending on (i) the variable structure (i.e., variables organised into a single block of variables or into several ones) and (ii) on the variable's nature (i.e., quantitative or qualitative values; or mixture of quantitative and qualitative values), different factor analyses could be applied. More precisely, PCA [23] was applied to numerical variables whereas MCA [15,24] was applied to nominal ones. In the case of a mixture of numerical and nominal variables, the FAMD method could be used [13]. When variables were organised into several blocks (e.g., variables measured at different times or variables organised into meaningful categories of characteristics such as IPAs, pesticides and nutritional resources), a MFA could be used [25].

These three indices (numerical, ordinal and nominal) summarised the rich information of eight IPAs quantified at two time points into synthetic values. They provided different yet complementary information. Similar to a toolbox, they could be chosen according to the aim pursued. The numerical index reflecting the coordinates on the first component was easy to calculate and implement in further statistical analyses since each site had a distinct value of exposure on a continuum. It could be easily included in all standard statistical analyses (i.e., regression). However, only a limited amount of the original information (when rich and multidimensional) was contained in this index. In our example, the 128 sampled sites were mostly characterised by the loads of DWV-B, SBV and *N. ceranae*. If the aim was to include the complex data on exposure to IPAs into further statistical analysis, the numerical index should be chosen bearing in mind that the information contained in this index was highly simplified when data are rich. In order to ease the interpretation of the numerical index, an ordinal index using quartiles of the coordinates on the first component was created. This ordinal index was much easier to interpret than the numerical one. However, it had the same limits in the case of multidimensional data: it summarised only a small amount of information contained in the data (20% of the information in our study). DWV-B, SBV and *N. ceranae* were also the discriminative IPAs that characterised low and high index values. In our example and for this index, each site belonged to a 4-level ordered scale of exposure. The amount of information summarised in the nominal index was the highest among the three types of indices (94%) but the index was less practical to use in subsequent statistical analyses as only a reduced number of statistical analyses are currently devoted to nominal data. If the biologist needed to interpret data with deep insight, the nominal index would be the most relevant tool. In our example, the dataset resulted in four distinct categories of exposure configuration, including the same antagonistic relationship between *N. ceranae* and DWV-B as observed in the numerical index (Appendix D). However, the information was completed with more nuances found for the remaining IPAs. These results support the previous hypothesis that variation in tolerance might be much more prevalent in systems with less pathogenic parasites (in our study: BQCV, DWV, SBV or *N. ceranae*) than in those with more pathogenic parasites (in our study: ABPV, CBPV or *N. apis*), and where the costs of tolerance are expressed not only in resistance [26]. In other words, less pathogenic IPAs were found more often than IPAs more lethal for their hosts. Therefore loads might be more relevant for assessing bee health. Our results were also consistent with hypotheses of an antagonistic relationship between *N. ceranae* and DWV in the absence of *Varroa destructor* mites [27]. Doublet et al. (2015) experimentally explored this hypothesis with oral infections of honey bees and found that the prior establishment of *N. ceranae* in midgut negatively affected the development of DWV; but the competition between both pathogens was asymmetric and the prior infection by DWV did not prevent the *N. ceranae* infection [28]. Contrary to country, focal crops were not significantly linked to IPA exposure in honey bees. Nevertheless, time of colony exposure on sites was linked to IPA exposure. There was an evolution in pathogen loads between installation of colonies on sites and end of the flowering bloom. One other study has also shown the impact of IPA exposure on variation of morphological traits in honey bees at site level [29]. Interestingly, crop surrounding sites also impacted this variation. To conclude, we proposed the calculation of three different types of indices to summarise a large number of variables collected by biologists. This calculation could be applied to a wild range of data; an example on bee data being given here. As factor analyses summarise the information contained in the overall data into components (the first one being the best summary into a single dimension, the first two ones into two dimensions, and so on) they are relevant tools for biologists to summarise their data. In addition, factor analyses are developed for various nature of variables (numerical, nominal, mixture of numerical and nominal) and various structures of variables (single or several blocks). Thus, these methods can be applied to all types of data to summarise them into indices.

The factor analysis helps the biologist to make a biological interpretation of a set of factors from the variable analyses. For honey bee heath, the field of applications of these synthetic indices is huge and includes risk assessment data. Bees are exposed to IPAs and pesticides, however these stressors do not present the same risk depending on quantity and type of IPAs (virus, bacteria or else) or pesticides (fungicides, insecticides or herbicides). Exposure data can be coupled with hazard data (toxicological data) to express risk data. The calculation of indices described in this paper can also be applied to datasets on the risks posed to honey bees by other stressors (nutrition, access to habitats, others). Indices can be used for further statistical analysis but can also be used by policy makers and public instances to characterize a given situation.

## Author contributions

1 Conceived and designed experiments: SB, MHTNN, ML, MPC.
2 Performed experiments: MHTNN, SB, ML, MPC.
3 Analysed and interpreted the data: MHTNN, AB, ED, MPR, ML, CD, SB, MPC.
4 Contributed reagents, materials, analysis tools or data: SB, MHTNN, AB, ED, CD, ML, MPC.
5 Wrote the paper: MHTNN, SB, ML, AB, ED, MPR, MPC.

## Funding

This work was supported by the European Union's Horizon 2020 research and innovation program under grant agreement No 773921.

## Additional information

No additional information is available for this paper.

## Declaration of competing interest

The authors declare that they have no known competing financial interests or personal relationships that could have appeared to influence the work reported in this paper.

## Figures and Tables

**Table A.1:**
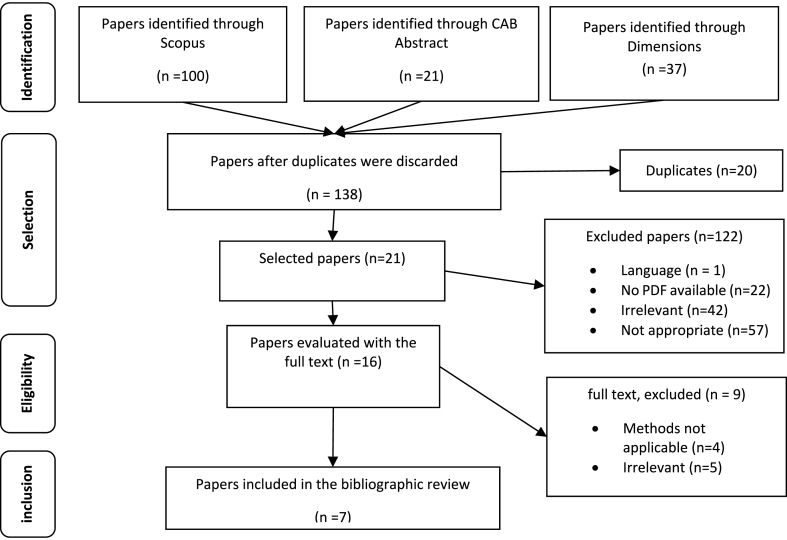
Results of the bibliographic analysis following PRISMA method to look for existing indices describing honey bee exposure to stressors [30].

**Fig. B.1:**
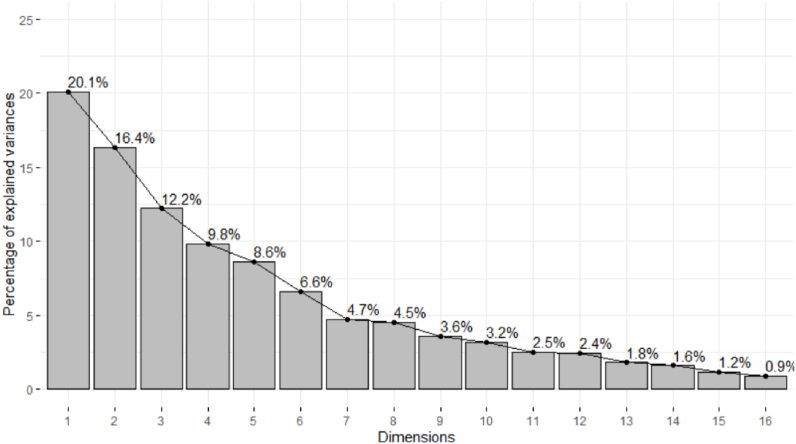
MFA scree plot of honey bee exposure to IPAs. Percentages are proportion of the total variances explained by each dimension.

**Table C.1:** The three types of indices calculated for each site to characterize honey bee exposure to IPAs.

Site identification	Numerical index	Ordinal index	Nominal index
GBR_OSR_01	0,99	4	D
GBR_OSR_02	1,29	4	D
GBR_OSR_03	2,17	4	D
GBR_OSR_04	2,12	4	D
GBR_OSR_05	1,88	4	D
GBR_OSR_06	1,8	4	D
GBR_OSR_07	1,28	4	D
GBR_OSR_08	1,94	4	D
GBR_APP_09	0	2	C
GBR_APP_10	0,68	3	C
GBR_APP_11	0,25	3	C
GBR_APP_12	0,73	3	C
GBR_APP_13	0,01	2	C
GBR_APP_14	−0,51	2	B
GBR_APP_15	0,66	3	C
GBR_APP_16	−0,3	2	B
ESP_OSR_01	−0,79	2	B
ESP_OSR_02	0,37	3	B
ESP_OSR_03	0,84	3	B
ESP_OSR_04	−1,18	1	B
ESP_OSR_05	−2,12	1	B
ESP_OSR_06	−1,67	1	B
ESP_OSR_07	−1,28	1	B
ESP_OSR_08	−0,32	2	B
ESP_APP_09	−0,64	2	B
ESP_APP_10	−1,12	1	B
ESP_APP_11	−1,79	1	B
ESP_APP_12	0,4	3	B
ESP_APP_13	−1,32	1	B
ESP_APP_14	−0,74	2	B
ESP_APP_15	−0,31	2	B
ESP_APP_16	−1,63	1	B
CHE_OSR_01	−0,05	2	C
CHE_OSR_02	0,53	3	C
CHE_OSR_03	0,57	3	C
CHE_OSR_04	0,05	2	C
CHE_OSR_05	0,38	3	C
CHE_OSR_06	1	4	C
CHE_OSR_07	1,41	4	C
CHE_OSR_08	0,43	3	C
CHE_APP_09	0,8	3	C
CHE_APP_10	1,32	4	C
CHE_APP_11	1,03	4	C
CHE_APP_12	0,81	3	C
CHE_APP_13	0,38	3	C
CHE_APP_14	0,13	2	C
CHE_APP_15	0,71	3	C
CHE_APP_16	0,51	3	C
ITA_OSR_01	1,66	4	C
ITA_OSR_02	0,73	3	C
ITA_OSR_03	0,26	3	C
ITA_OSR_04	0,15	2	C
ITA_OSR_05	1,3	4	C
ITA_OSR_06	0,71	3	C
ITA_OSR_07	2,07	4	C
ITA_OSR_08	0,77	3	C
ITA_APP_09	1,85	4	C
ITA_APP_10	1,54	4	C
ITA_APP_11	1,93	4	C
ITA_APP_12	0,27	3	C
ITA_APP_13	1,87	4	C
ITA_APP_14	1,21	4	C
ITA_APP_15	0,51	3	C
ITA_APP_16	0,91	3	C
GER_OSR_01	−0,79	2	C
GER_OSR_02	−0,07	2	C
GER_OSR_03	0,23	3	C
GER_OSR_04	0,34	3	C
GER_OSR_05	−0,91	2	A
GER_OSR_06	0,11	2	C
GER_OSR_07	−0,02	2	C
GER_OSR_08	−0,56	2	C
GER_APP_09	0,24	3	C
GER_APP_10	0,86	3	C
GER_APP_11	−0,75	2	C
GER_APP_12	0,49	3	C
GER_APP_13	0,18	2	C
GER_APP_14	1	4	C
GER_APP_15	0,4	3	C
GER_APP_16	−0,68	2	C
IRL_OSR_01	−1,34	1	A
IRL_OSR_02	−3,58	1	A
IRL_OSR_03	−2,35	1	A
IRL_OSR_04	−2,63	1	A
IRL_OSR_05	−0,91	2	A
IRL_OSR_06	−1,64	1	A
IRL_OSR_07	−2,81	1	A
IRL_OSR_08	−2,34	1	A
IRL_APP_09	−2,41	1	A
IRL_APP_10	−1,96	1	A
IRL_APP_11	−0,91	2	A
IRL_APP_12	−1,33	1	A
IRL_APP_13	−1,92	1	A
IRL_APP_14	−1,69	1	A
IRL_APP_15	−2,26	1	A
IRL_APP_16	−1,54	1	A
EST_OSR_01	2,32	4	D
EST_OSR_02	1,8	4	D
EST_OSR_03	1,8	4	C
EST_OSR_04	1,69	4	D
EST_OSR_05	−0,61	2	A
EST_OSR_06	1,34	4	C
EST_OSR_07	0,65	3	C
EST_OSR_08	1,83	4	D
EST_APP_09	−1,63	1	A
EST_APP_10	0,66	3	D
EST_APP_11	1,43	4	D
EST_APP_12	1,47	4	D
EST_APP_13	1,89	4	D
EST_APP_14	0,95	3	C
EST_APP_15	1,46	4	D
EST_APP_16	1,86	4	D
SWE_OSR_01	−1,48	1	B
SWE_OSR_02	−1,54	1	B
SWE_OSR_03	−1,09	2	B
SWE_OSR_04	−1,07	2	B
SWE_OSR_05	0,06	2	B
SWE_OSR_06	−2,05	1	B
SWE_OSR_07	−0,37	2	B
SWE_OSR_08	−0,46	2	B
SWE_APP_09	−0,36	2	B
SWE_APP_10	−1,26	1	B
SWE_APP_11	−0,72	2	B
SWE_APP_12	−1,25	1	B
SWE_APP_13	−1,36	1	B
SWE_APP_14	−1,24	1	B
SWE_APP_15	−1,19	1	B
SWE_APP_16	−1,44	1	B

**Table D.1:** Contingency table with the number of sites reported for ordinal and nominal indices. The ordinal index classes are horizontal (from 1 to 4), the nominal index classes being vertical (A to D).

ordinal ind.	1	2	3	4
nominal ind.				
A	15	4	0	0
B	17	14	3	0
C	0	14	28	15
D	0	0	1	18

## Appendix C

The same dataset was summarised in three different ways. The numerical index provides a numerical value for each site on a continuum. The ordinal and the nominal indices provide four site clusters (continuous clusters 1, 2, 3 and 4 for the ordinal index and distinctive clusters A, B, C and D for the nominal index).

## Appendix D

From this table, it follows that the nominal-cluster A was mainly associated with the ordinal-cluster 1 (low values of SBV and *N. ceranae*). The nominal-cluster B was associated with the ordinal-clusters 1 and 2 while the nominal-cluster C was associated with the ordinal-clusters 3 (high values of *N. ceranae*) and to a lesser extent with 2 and 4. The nominal-cluster D was associated with the ordinal-cluster 4 (high values of DWV-B and *N. ceranae*).
